# Incidence and Progression of Acute Kidney Injury Following Head and Neck Surgeries for COVID-19-Associated Mucormycosis: A Prospective Observational Study

**DOI:** 10.7759/cureus.116469

**Published:** 2026-09-18

**Authors:** Kalyani M Rapeti, Habib Md R Karim, Chinmaya K Panda, Neha Singh, TC Arun, Ripudaman Arora

**Affiliations:** 1 Anesthesiology, Critical Care, and Pain, Homibhabha Cancer Hospital and Research Centre, Visakhapatnam, IND; 2 Anesthesiology, Critical Care, and Pain Medicine, All India Institute of Medical Sciences, Guwahati, Guwahati, IND; 3 Anesthesiology and Critical Care, All India Institute of Medical Sciences, Raipur, Raipur, IND; 4 Anaesthesiology, Medanta Hospital, Patna, IND; 5 Otorhinolaryngology, All India Institute of Medical Sciences, Raipur, Raipur, IND

**Keywords:** acute kidney injury, covid-19, kidney disease progression, mucormycosis, risk factors

## Abstract

Background: COVID-19-associated mucormycosis (CAM) surged because of the combination of systemic corticosteroids, poorly controlled diabetes mellitus, and altered host immunity. Rhino-orbito-cerebral mucormycosis (ROCM) requires extensive surgical debridement and systemic nephrotoxic liposomal amphotericin B (L-AMB) therapy. This study evaluated the incidence, temporal course, and association of postoperative acute kidney injury (AKI) with various clinical factors in this high-risk surgical cohort.

Materials and methods: This prospective observational cohort study enrolled 49 adult patients who underwent major head and neck surgical debridement for ROCM and required postoperative critical care management. We excluded patients with established renal failure. We performed and recorded serial clinical and biochemical monitoring daily over the next five days. AKI incidence and severity were staged according to Acute Kidney Injury Network (AKIN) criteria.

Results: A total of 49 patients were evaluated for AKI, of which 9 developed AKI (18.4%). Patients who developed AKI had a higher mean age (56.6 ± 10.0 years versus 49.4 ± 10.3 years) and preoperative serum creatinine (1.1 ± 0.5 vs 0.9 ± 0.2 mg/dL), but these differences were not statistically significant. However, patients who developed post-operative AKI had a significantly lower estimated glomerular filtration rate (eGFR) (87.4 ± 27.1 vs 102.9 ± 17.4 mL/min/1.73 m²; p = 0.03), and a significantly higher prevalence of AKI was found in patients having pre-existing hypertension (77.8% vs. 32.5%; p = 0.02). Conversely, mean duration (p = 0.35), cumulative preoperative L-AMB dose (p = 0.319), and background diabetes mellitus (p = 0.136) did not differ significantly between groups.

Conclusion: AKI was noted in nearly one-fifth of patients after ROCM surgery and was more common in older patients with hypertension and poorer baseline renal function. Baseline renal function appeared more important than cumulative L-AMB exposure or diabetes, highlighting the need for early renal risk assessment and close postoperative monitoring.

## Introduction

The coronavirus disease 2019 (COVID-19) pandemic precipitated an unprecedented surge in COVID-19-associated mucormycosis (CAM), particularly in India, where widespread corticosteroid use, poorly controlled diabetes mellitus, and altered host immunity created a fertile substrate for invasive fungal infection [1,2]. Mucormycosis is caused by fungi of the order Mucorales and is characterized by aggressive angioinvasion, rapid tissue necrosis, and high mortality if untreated [3]. Among its clinical subtypes, rhinoorbitocerebral mucormycosis (ROCM) is the predominant form in the Indian subcontinent, accounting for most CAM cases reported during the second wave of the pandemic [4].

Management of ROCM mandated aggressive surgical debridement of involved tissues combined with systemic antifungal therapy [5]. Liposomal amphotericin B (L-AMB) remains the antifungal agent of choice; however, it has a well-recognized propensity for nephrotoxicity through direct tubular injury, renal vasoconstriction, and alterations in tubular permeability. In a retrospective analysis by Yamazaki et al., renal dysfunction was documented in a significant proportion of patients receiving L-AMB for hematological disorders, with hypokalemia emerging as a concomitant electrolyte complication [6]. Takazono et al. identified prior use of ACE inhibitors or carbapenems, concomitant administration of catecholamines or immunosuppressants, and pre-existing hypokalemia as independent predictors of L-AMB-associated AKI in a real-world clinical cohort [7]. However, potassium supplementation failed to confer benefit at any stage of AKI in patients with hypokalemia receiving L-AMB [8].

Beyond antifungal nephrotoxicity, the perioperative period itself constitutes a significant risk window for acute kidney injury (AKI). The Acute Kidney Injury Network (AKIN) criteria define AKI as a rise in serum creatinine ≥0.3 mg/dL or ≥50% from baseline within 48 hours, or urine output <0.5 mL/kg/hr for >6 hours despite adequate fluid resuscitation, and have been validated and found to have a strong relationship between the stages and the need for dialysis and mortality [9].

Although patient outcomes are evaluated in both the short and long term, data on AKI incidence and progression during the initial ICU days in such cases are scarce. It is pertinent to note that the incidence rises substantially in higher-acuity surgical settings, reaching 49% in cardiac surgery requiring bypass and 31% to 43% in postoperative head and neck cancer patients on chemoradiotherapy, with some patients progressing to stage III requiring dialysis [10,11]. Wang et al. found that postoperative AKI was associated with a fourfold increase in the odds of mortality [12]. Further, chronic kidney disease (CKD) is increasingly recognized as a precursor to postoperative AKI, with long-term follow-up data confirming a substantially elevated risk of end-stage renal disease among AKI survivors [13].

In patients with CAM undergoing head and neck debridement, the risk of AKI is uniquely amplified by the convergence of multiple concurrent insults: prolonged L-AMB therapy initiated preoperatively; the hemodynamic perturbations and systemic inflammatory response associated with extensive surgical debridement; perioperative electrolyte disturbances, particularly hypokalemia and hypomagnesemia; and a high background prevalence of diabetes mellitus and antihypertensive medication use [5-8]. Despite this convergence of risk factors, the incidence and temporal trajectory of AKI specifically in the postoperative CAM population have not been systematically characterized.

This prospective observational study was therefore conducted to determine the incidence of AKI in patients admitted to the critical care unit following head and neck surgeries for COVID-19-associated ROCM, delineate the temporal course of renal dysfunction during the early postoperative period, and identify the clinical and pharmacological factors associated with its development.

## Materials and methods

After obtaining Institutional Review Board approval (AIIMSRPR/RC(P)/2021/362), the study was registered with the Clinical Trials Registry- India (CTRI/2022/01039200). This prospective observational study was conducted in the postoperative intensive care unit of a tertiary care hospital. The study was conducted in accordance with the Declaration of Helsinki.

The patients were enrolled after obtaining informed consent, either from the patients themselves or from their legally authorized representatives. The inclusion and exclusion criteria are mentioned in Table 1. Participants were enrolled in this study from June 2021 to November 2021. 

**Table 1:** Enrollment criteria of the participants COVID-19: coronavirus disease of 2019; eGFR: estimated glomerular filtration rate; ROCM: rhino-oculo-cerebro-mucormycosis.

Inclusion Criteria	Exclusion Criteria
Age: 18 years and above	Refusal to participate
COVID-19-associated ROCM	Known chronic kidney disease
Undergoing surgery under general anesthesia	Preoperative creatinine >2 mg/dL
Required postoperative critical care	Preoperative eGFR less than 60 mL/min
Remained in observation for at least 5 days	Patients on hemodialysis

The same group of surgeons, anesthesiologists, and intensive care team provided perioperative care. Intraoperatively, hemodynamics were targeted to maintain a mean arterial pressure (MAP) of 60-65 mmHg. We administered adequate fluid therapy to maintain euvolemia and used vasopressors to maintain the target MAP. We recorded serum creatinine, blood urea levels, and serum electrolytes preoperatively, along with urine output intraoperatively and from postoperative day (POD) 1 to 5. We evaluated CAM-AKI incidence and severity using the AKIN criteria and expressed them as absolute numbers and percentages. We also noted surgery duration and blood loss.

We analyzed temporal trends in serum creatinine, blood urea levels, serum electrolytes, and urine output across PODs and CAM-AKI status. We analyzed data using an independent-samples t-test and a chi-square test in IBM SPSS Statistics for Windows, Version 25 (Released 2017; IBM Corp., Armonk, New York). We performed univariate analysis to identify risk factors for AKI. We compared paired data using a paired t-test and analyzed independent data using an unpaired t-test.

## Results

A total of 49 patients were enrolled and studied for the incidence of AKI. They ranged in age from 30 to 74 years, with 36 (73.47%) male and 13 (26.53%) female participants. A total of 9 patients (18.37%) met the AKIN criteria during the observation window. Patients who developed AKI had a significantly higher mean age than the non-AKI group (p = 0.07), and the incidence proportion increased significantly with advancing age, peaking in the 50-59 years age group (44.4% of incidence; Table 2).

Although the study group comprised only 13 females, with male predominance overall, gender distribution did not differ significantly between groups (p = 0.683). No significant association was found with anthropometric parameters. Diabetes mellitus was highly prevalent in AKI patients, 8 (88.9%) vs 24 (60%) in the non-AKI group (p = 0.136), but the difference did not reach statistical significance. The AKI group had a significantly higher incidence of hypertension, 7 (77.8%), compared with the non-AKI group, 13 (32.5%), with statistical significance (p = 0.02). The mean cumulative dose of L-AmB and duration of therapy did not differ significantly between groups, and baseline vital parameters were also similar between groups (p > 0.05 for all comparisons) (Table 2). 

**Table 2:** Baseline demographic and clinical characteristics of AKI and non-AKI groups compared using independent sample t-test Two-sided p-values are calculated assuming equal variances. AKI: acute kidney injury; SD: standard deviation; BMI: body mass index; CI: confidence interval; DBP: diastolic blood pressure; df: degrees of freedom; L-AMB: liposomal amphotericin B; SBP: systolic blood pressure; SE: standard error; SpO₂: peripheral oxygen saturation.

Variable	AKI status	Mean (SD)	t-statistic	df	Two-sided p	Mean difference	SE difference	95% CI
Age (years)	Yes	56.6 (10.0)	1.89	47	0.07	7.13	3.78	-0.47 to 14.73
	No	49.4 (10.3)	1.92	12.13		7.13	3.71	-0.93 to 15.19
Height (cm)	Yes	162.2 (9.0)	0.34	47	0.74	0.82	2.44	-4.09 to 5.74
	No	161.4 (6.0)	0.26	9.67		0.82	3.15	-6.23 to 7.88
Weight (kg)	Yes	67 (8.8)	1.29	47	0.20	3.68	2.85	-2.06 to 9.41
	No	63.3 (7.5)	1.16	10.73		3.68	3.18	-3.34 to 10.69
BMI (kg/m^2^)	Yes	25.5 (3.2)	1.13	47	0.26	1.25	1.1	-0.97 to 3.47
	No	24.2 (3.0)	1.08	11.31		1.25	1.16	-1.29 to 3.79
Therapy before surgery (days)	Yes	2.98(1.42)	-0.37	47	0.71	-0.2	0.53	-1.26 to 0.87
	No	2.78(1.48)	-0.36	11.57		-0.2	0.54	-1.38 to 0.99
Heart rate (beats/minute)	Yes	82.8 (8.3)	-0.23	47	0.82	-0.77	3.38	-7.58 to 6.04
	No	83.6 (9.3)	-0.25	12.99		-0.77	3.14	-7.55 to 6.01
SBP (mmHg)	Yes	128.8 (15.8)	1.36	47	0.18	7.85	5.76	-3.74 to 19.45
	No	120.9 (15.6)	1.35	11.79		7.85	5.81	-4.83 to 20.53
DBP (mmHg)	Yes	81.6 (8.9)	0.97	47	0.34	3.71	3.83	-3.99 to 11.40
	No	77.9 (10.6)	1.09	13.73		3.71	3.4	-3.61 to 11.02
SpO₂ (%)	Yes	98.0 (2.2)	-1.08	47	0.29	-0.63	0.58	-1.79 to 0.54
	No	98.6 (1.4)	-0.82	9.56		-0.63	0.76	-2.33 to 1.08
L-AMB (dose in mg)	Yes	528.3 (209.5)	0.5	47	0.62	31.83	63.66	-96.24 to 159.9
	No	496.5 (164.0)	0.43	10.32		31.83	74.49	-133.45–197.11

AKI patients showed a nonsignificant trend toward higher preoperative serum creatinine levels (1.1 ± 0.5 vs 0.9 ± 0.2 mg/dL; p = 0.06) but significantly lower eGFR values (87.4 ± 27.1 vs 102.9 ± 17.4 mL/min/1.73 m²; p = 0.03); urea, urine output, sodium, potassium, chloride, and magnesium did not differ significantly, indicating that impaired baseline renal function was associated with subsequent AKI development (Table 3). 

**Table 3:** Preoperative renal and electrolyte parameters in AKI and non-AKI groups compared using independent-samples t-test Two-sided p-values are calculated assuming equal variances. CI: confidence interval; df: degrees of freedom; eGFR: estimated glomerular filtration rate; SE: standard error; t: t-statistic

Variable	AKI status	Mean (SD)	t	df	Two-sided p-values	Mean difference	SE difference	95% CI
Urea	Yes	20.33 (9.96)	0.775	47	0.44	2.16	2.78	-3.44 to 7.76
	No	18.18 (6.95)	0.61747	9.82		2.16	3.5	-5.65 to 9.97
Creatinine	Yes	1.1 (0.5)	1.95	47	0.06	0.21	0.11	-0.01 to 0.43
	No	0.9 (0.2)	1.17	8.59		0.21	0.18	-0.20 to 0.62
Baseline eGFR	Yes	87.4 (27.1)	-2.16	47	0.03	-15.44	7.16	-29.85 to -1.03
	No	102.9 (17.4)	-2.04	47		-15.44	7.57	-32.05 to 1.17
Potassium	Yes	3.4 (0.6)	-0.85	47	0.4	-0.17	0.2	-0.59 to 0.24
	No	3.6 (0.5)	-0.82	11.39		-0.17	0.21	-0.64 to 0.29
Magnesium	Yes	1.7 (0.3)	-0.9	47	0.37	-0.12	0.13	-0.38 to 0.15
	No	1.7 (0.2)	-1.02	13.74		-0.12	0.12	-0.37 to 0.13
Platelet count	Yes	315.7 (121.4)	-0.42	47	0.68	-17.86	43.07	-104.51 to 68.79
	No	297.8 (90.25)	-0.5	15.33		-17.86	35.69	-93.80 to 58.07
Neutrophils	Yes	67.7 (12.15)	0.36	47	0.72	1.52	4.2	-6.92 to 9.97
	No	69.36 (6.36)	0.53	23.28		1.52	2.86	-4.40 to 7.45

Patients who developed AKI had a higher mean intraoperative blood loss than those without AKI (700.0 ± 220.4 mL vs. 657.5 ± 162.7 mL), but the difference was not statistically significant (t = 0.654, df = 46, p = 0.516). Similarly, the mean duration of surgery was longer in patients with AKI than in those without AKI (165.6 ± 38.2 vs. 152.1 ± 41.4 minutes), although this difference was also not statistically significant (t = 0.884, df = 46, p = 0.381).

Serial post-treatment values for urea, creatinine, urine output, sodium, potassium, chloride, magnesium, and eGFR were compared with preoperative baseline values on Days 1-5. A significant increase in urea was observed from Day 1 to Day 5, suggesting a transient but significant deterioration in renal function following treatment. Creatinine also rose significantly on Days 1 and 2. Sodium showed no significant changes at any point. Potassium and chloride fell significantly, while magnesium rose significantly throughout. eGFR values fell significantly on Days 1 and 2 and then recovered to baseline by Days 3-5. These findings indicate a transient deterioration in renal function and electrolyte balance in the early post-treatment period, with recovery toward baseline by Days 3-5 for most parameters (Table 4).

**Table 4:** Day-wise comparison of renal and electrolyte parameters with pre-operative baseline tested using paired t-test CI: confidence interval; df: degrees of freedom; eGFR: estimated glomerular filtration rate; K: potassium; Mg: magnesium; Na: sodium; p: p-value; pre-op: preoperative; SE: standard error; t: t-statistic; UOP: urine output.

Matched parameter pairings	t	df	Two-sided p	Mean difference	SE difference	95% Confidence interval (CI)
Pre-op urea vs. Day 1 urea	-2.88	48	0.01	-4.16	1.44	-7.06 to -1.27
Pre-op urea vs. Day 2 urea	-1.98	48	0.05	-4.14	2.09	-8.34 to 0.06
Pre-op urea vs. Day 3 urea	-0.68	48	0.50	-0.92	1.34	-3.61 to 1.77
Pre-op urea vs. Day 4 urea	-1.15	48	0.25	-1.63	1.42	-4.48– 1.22
Pre-op urea vs. Day 5 urea	-1.46	48	0.15	-2.51	1.71	-5.95 to 0.93
Pre-op creatinine vs. Day 1 creatinine	-3.01	48	0.004	-0.12	0.04	-0.20– -0.04
Pre-op creatinine vs. Day 2 creatinine	-3.24	48	0.002	-0.16	0.05	-0.21 to -0.06
Pre-op creatinine vs. Day 3 creatinine	0.3	48	0.76	0.01	0.03	-0.05 to 0.06
Pre-op creatinine vs. Day 4 creatinine	0.46	48	0.65	0.01	0.03	-0.04 to 0.06
Pre-op creatinine vs. Day 5 creatinine	0.26	48	0.79	0.01	0.03	-0.05– 0.07
Pre-op eGFR vs. Day 1 eGFR	2.58	48	0.01	6.54	2.53	-1.45 to 11.62
Pre-op eGFR vs. Day 2 eGFR	2.91	48	0.01	9.02	3.10	-2.78– 15.24
Pre-op eGFR vs. Day 3 eGFR	-0.15	48	0.88	-0.33	2.17	-4.69 to 4.03
Pre-op eGFR vs. Day 4 eGFR	-0.17	48	0.87	-0.46	2.75	-5.99 to 5.08
Pre-op eGFR vs. Day 5 eGFR	-0.05	48	0.96	-0.10	2.05	-4.21 to 4.01
Pre-op UOP vs. Day 1 UOP	1.75	48	0.09	181.43	103.45	-26.57 to 389.4
Pre-op UOP vs. Day 2 UOP	0.63	48	0.53	69.29	110.54	-152.9 to 291.53
Pre-op UOP vs. Day 3 UOP	0.38	48	0.71	39.29	103.98	-169.77– 248.34
Pre-op UOP vs. Day 4 UOP	1.51	48	0.14	152.65	101.09	-50.59– 355.90
Pre-op UOP vs. Day 5 UOP	1.33	48	0.19	166.27	124.86	-84.78 to 417.31
Pre-op Na vs. Day 1 Na	-0.72	48	0.47	-0.92	1.27	-3.47 to 1.64
Pre-op Na vs. Day 2 Na	-0.74	48	0.46	-0.98	1.33	-3.65 to 1.69
Pre-op Na vs. Day 3 Na	-1.00	48	0.32	-1.39	1.39	-4.184 to 1.41
Pre-op Na vs. Day 4 Na	-1.48	48	0.14	-1.96	1.32	-4.61 to 0.69
Pre-op Na vs. Day 5 Na	-1.56	48	0.12	-1.96	1.25	-4.48 to 0.56
Pre-op K vs. Day 1 K	0.41	48	0.68	0.03	0.08	-0.13 to 0.19
Pre-op K vs. Day 2 K	1.51	48	0.14	0.10	0.07	-0.03 to 0.23
Pre-op K vs. Day 3 K	2.15	48	0.04	0.16	0.08	0.01 to 0.32
Pre-op K vs. Day 4 K	3.55	48	0.001	0.24	0.07	0.10 to 0.37
Pre-op K vs. Day 5 K	1.63	48	0.11	0.13	0.08	-0.03 to 0.30
Pre-op chloride vs. Day 1 chloride	-0.08	48	0.94	-0.04	0.54	-1.12 to 1.04
Pre-op chloride vs. Day 2 chloride	1.62	48	0.11	0.88	0.54	-0.21 to 1.97
Pre-op chloride vs. Day 3 chloride	1.30	48	0.20	0.61	0.47	-0.34 to 1.56
Pre-op chloride vs. Day 4 chloride	-3.62	48	0.001	-1.63	0.45	-2.54 to -0.73
Pre-op chloride vs. Day 5 chloride	-0.84	48	0.41	-0.47	0.56	-1.59 to 0.66
Pre-op Mg vs. Day 1 Mg	-1.23	48	0.22	-0.04	0.04	-0.12 to 0.03
Pre-op Mg vs. Day 2 Mg	-2.56	48	0.01	-0.09	0.04	-0.16 to -0.02
Pre-op Mg vs. Day 3 Mg	-3.94	48	0.0003	-0.14	0.04	-0.21 to -0.07
Pre-op Mg vs. Day 4 Mg	-4.12	48	0.0001	-0.15	0.04	-0.22 to -0.07
Pre-op Mg vs. Day 5 Mg	-6.29	48	<0.0001	-0.21	0.03	-0.28 to -0.15

Based on postoperative changes in serum creatinine, acute kidney injury was assessed using the Acute Kidney Injury Network (AKIN) criteria. Among the 49 study participants, 9 (18.4%) patients developed AKI. Stage 1 AKI was observed in 8 (16.3%) patients and Stage 2 AKI in 1 (2.0%) patient. No patient developed Stage 3 AKI. The majority of AKI cases were therefore mild in severity, corresponding to AKIN Stage 1.

## Discussion

At the time of the study's inception and during the COVID-19 pandemic, data on AKI incidence and progression were unavailable, particularly regarding the immediate impact and AKI trajectories in intensive care. One of our researchers (Karim HMR) studied AKI incidence and progression during intensive care stay in a cardiac surgical population, and we wanted to explore the same in CAM patients [10]. Such data help us understand patients' trajectories. Our study found that most AKIs were stage I and self-limiting, similar to Karim et al.'s findings [10]. Karim et al. also found that only stage III AKI progressed to the need for hemodialysis [10]. However, we could not confirm this in our cohort, as no stage III AKI cases were observed.

The ROCM cohort on L-AMB therapy in our study had an AKI incidence of 18.4%, which was relatively higher than that in most major surgeries but lower than that in head and neck cancer surgeries with concurrent chemoradiation with cisplatin [11]. This incidence rate is substantially higher than the non-cardiac postoperative AKI incidence, likely reflecting the cumulative burden of nephrotoxicity from co-therapy with L-AMB and hemodynamic factors. A meta-analysis across surgical ICUs reported an AKI incidence of approximately 16% (95% CI, 14%-17%) in patients undergoing abdominal surgery [14]. This incidence is relatively conservative compared with some surgical ICU cohorts reporting 53.4% (95% CI, 48.4%-58.4%) [15]. Our low incidence might reflect exclusion criteria that excluded patients with pre-existing CKD and those undergoing other higher-acuity surgeries, as well as a cohort with a smaller elderly population [16]. The meta-analysis also suggested a preventive role of individualized BP management [14]. We, however, maintained BP with a target MBP of 65 mmHg or above.

The higher mean age in the AKI cohort than in the non-AKI group (56.6 vs 49.4 years) showed a borderline, nonsignificant trend toward predicting AKI (p = 0.07). This is consistent with supporting literature indicating that age-related reductions in nephron numbers, impaired autoregulatory capacity, and reduced physiological resilience to hemodynamic stress make older adults more vulnerable to postoperative renal injuries [16]. Similar age-related findings have also been observed in laboratory-confirmed COVID-19 infections among ICU admissions, as well as in non-COVID surgical populations [17,18].

Hypertension is an independent risk factor for major adverse events in surgical ICU patients, and hypertensive patients who develop AKI are at higher risk of fatal events [19]. Perioperative BP-lowering drugs, such as angiotensin-converting enzyme (ACE) inhibitors, angiotensin receptor blockers, and calcium channel blockers, are also more effective in reducing BP in hypertensive patients but are associated with higher complication rates [19]. Renal autoregulation is impaired due to chronic hypertension, which causes an increased incidence of postoperative AKI due to intraoperative vasodilatation, blood loss, and hemodynamic instability, which further causes poor postoperative outcomes [15,19]. Our cohort also showed that AKI was significantly more common in patients with pre-existing hypertension (35.0% (7/20)) than in non-hypertensives (6.9% (2/29); p = 0.022).

Diabetes mellitus was present in 88.9% of AKI patients and 60.0% of non-AKI patients in our cohort. Unlike Wubet et al., our finding did not reach statistical significance [15]. However, the prevalence of uncontrolled diabetes among CAM patients was higher, which correlates with the epidemiological data, especially in India [20]. Further, COVID-19 patients also received steroids as part of treatment, which also causes hyperglycemia. In our cohort, baseline eGFR was a significant indicator of AKI susceptibility (p = 0.03), whereas baseline serum creatinine did not differ significantly (p = 0.06). This discrepancy underscores that eGFR provides a more sensitive depiction of true baseline renal reserve than raw, unadjusted creatinine values, which are easily confounded by non-renal factors. While a recent postoperative AKI study found that higher baseline creatinine independently predicted AKI (p < 0.001) [17], our findings suggest that when raw creatinine does not reach statistical significance, calculating eGFR can unmask subclinical renal impairment that drives subsequent postoperative injury.

Similarly, neither cumulative dose (p = 0.319) nor duration of therapy (p = 0.35) showed an association with AKI in the present study, despite the known nephrotoxicity of L-AMB. Caution is warranted when interpreting this result, as the postoperative follow-up was only five days. Higher AKI incidence with extended L-AMB therapy was reported [21,22].

Serum potassium significantly changed on postoperative Day 3 (3.57 ± 0.5 mEq/L vs 3.41 ± 0.6 mEq/L at baseline; p = 0.018). This mechanism may reflect hypomagnesemia, as amphotericin B forms transmembrane pores in the distal renal tubule, leading to wasting and a self-reinforcing cycle of tissue damage that often precedes GFR decline [22]. Adequate potassium and magnesium supplementation during L-AMB therapy might disrupt this mechanism [22]. However, the evidence is limited, and supplementation should start before L-AMB initiation rather than after [8]. Furthermore, a >10% fall in potassium, as indicated by the hypokalemia index rather than single-timepoint values, serves as a sensitive surrogate biomarker for predicting AKI in patients receiving L-AMB [23]. Given the borderline baseline potassium in AKI patients in our study (mean 3.4 mEq/L), these findings reinforce the importance of strict potassium monitoring and proactive supplementation in ROCM patients receiving L-AMB to reduce L-AMB-related nephrotoxicity. Serum magnesium decreased progressively and significantly from Day 2 onward (Days 3 and 5: p = 0.001). Similar to potassium wasting, amphotericin B classically causes hypomagnesemia via the same pore-mediated distal tubular mechanism [22]. Guidelines recommend routine magnesium and potassium supplementation to mitigate these electrolyte losses with L-AMB use [22].

Our study also had notable limitations, including its single-center design and relatively small sample size. Treatment of COVID-19 patients kept changing as new evidence emerged, and we did not account for these factors. Further, we relied solely on urine output and creatinine levels, whereas using biomarkers might have altered the incidence or course. Multivariate analysis was not feasible due to limited data. Nevertheless, rhino-orbito-cerebral mucormycosis is a rare disease; thus, these results may not generalize to other major surgeries.

## Conclusions

Postoperative AKI is a frequent complication, accounting for nearly one in five patients (18.4%) undergoing extensive head and neck debridement for CAM-ROCM while on L-AMB therapy. Most cases were stage I or II AKI; the clinical course was self-limiting, characterized by a sharp drop in glomerular filtration within the first two postoperative days, followed by recovery to baseline from Day 3 onward. The early renal impairment was not temporally associated with preoperative exposure to liposomal amphotericin B or with glycemic status, even when these were appropriately monitored and managed. Instead, the occurrence of AKI was associated with baseline patient characteristics such as advanced age, pre-existing chronic hypertension, and depleted preoperative renal reserve.
